# Differences in Health-Related Quality of Life among Patients with Heart Failure

**DOI:** 10.3390/medicina60010109

**Published:** 2024-01-06

**Authors:** Ioannis Ventoulis, Vasileios Kamperidis, Maria Roselle Abraham, Theodore Abraham, Antonios Boultadakis, Efthymios Tsioukras, Aikaterini Katsiana, Konstantinos Georgiou, John Parissis, Effie Polyzogopoulou

**Affiliations:** 1 Department of Occupational Therapy, University of Western Macedonia, Keptse Area, 50200 Ptolemaida, Greece; tsioukefth@gmail.com (E.T.); akatsiana@uowm.gr (A.K.); kgeorgiou@uowm.gr (K.G.); 2 First Department of Cardiology, AHEPA University Hospital, Aristotle University of Thessaloniki, St Kiriakidi 1, 54636 Thessaloniki, Greece; vkamperidis@outlook.com; 3 Hypertrophic Cardiomyopathy Center of Excellence, University of California, San Francisco, CA 94117, USA; roselle.abraham@ucsf.edu (M.R.A.); theodore.abraham@ucsf.edu (T.A.); 4 Emergency Medicine Department, Attikon University Hospital, National and Kapodistrian University of Athens, Rimini 1, Chaidari, 12462 Athens, Greece; boult_doc@yahoo.gr (A.B.); jparissis@yahoo.com (J.P.); effiepol@med.uoa.gr (E.P.)

**Keywords:** health-related quality of life, heart failure, differences, quality of life, health status, patient-reported outcomes

## Abstract

Heart failure (HF) is characterized by a progressive clinical course marked by frequent exacerbations and repeated hospitalizations, leading to considerably high morbidity and mortality rates. Patients with HF present with a constellation of bothersome symptoms, which range from physical to psychological and mental manifestations. With the transition to more advanced HF stages, symptoms become increasingly more debilitating, interfere with activities of daily living and disrupt multiple domains of life, including physical functioning, psychological status, emotional state, cognitive function, intimate relationships, lifestyle status, usual role activities, social contact and support. By inflicting profuse limitations in numerous aspects of life, HF exerts a profoundly negative impact on health-related quality of life (HRQOL). It is therefore not surprising that patients with HF display lower levels of HRQOL compared not only to the general healthy population but also to patients suffering from other chronic diseases. On top of this, poor HRQOL in patients with HF becomes an even greater concern considering that it has been associated with unfavorable long-term outcomes and poor prognosis. Nevertheless, HRQOL may differ significantly among patients with HF. Indeed, it has consistently been reported that women with HF display poorer HRQOL compared to men, while younger patients with HF tend to exhibit lower levels of HRQOL than their older counterparts. Moreover, patients presenting with higher New York Heart Association (NYHA) functional class (III–IV) have significantly more impaired HRQOL than those in a better NYHA class (I–II). Furthermore, most studies report worse levels of HRQOL in patients suffering from HF with preserved ejection fraction (HFpEF) compared to patients with HF with reduced ejection fraction (HFrEF) or HF with mildly reduced ejection fraction (HFmrEF). Last, but not least, differences in HRQOL have been noted depending on geographic location, with lower HRQOL levels having been recorded in Africa and Eastern Europe and higher in Western Europe in a recent large global study. Based on the observed disparities that have been invariably reported in the literature, this review article aims to provide insight into the underlying differences in HRQOL among patients with HF. Through an overview of currently existing evidence, fundamental differences in HRQOL among patients with HF are analyzed based on sex, age, NYHA functional class, ejection fraction and geographic location or ethnicity.

## 1. Introduction

Heart failure (HF) is a complex, heterogeneous and dynamically evolving clinical syndrome, which is characterized by the inability of the heart to adequately serve as a blood pump due to underlying structural or functional abnormalities. It encompasses a constellation of symptoms and signs, which are attributed to elevated cardiac filling pressures and reduced cardiac output and are dictated by the activation of several pathophysiological mechanisms. Conventionally, HF has been divided into distinct subtypes according to left ventricular ejection fraction (EF); that is, HF with reduced ejection fraction (HFrEF) when EF is ≤40%, HF with mildly reduced ejection fraction (HFmrEF) when EF is between 41% and 49% and HF with preserved ejection fraction (HFpEF) when EF is ≥50% [1,2,3,4]. Regardless of the aforementioned classification, the clinical course of HF is gradual, yet non-linear, invariably leading to a progressive deterioration of the patient’s clinical status despite optimal medical therapy. This course is marked by frequent exacerbations and repeated hospitalizations due to increasingly more episodes of acute HF decompensation, which inevitably exert negative effects on the patient’s health-related quality of life (HRQOL) [5,6].

HRQOL assesses health status directly from the patient’s perspective, and not through the lens of the clinician, and reflects the multidimensional impact of a certain disease and its treatment on various levels of the patient’s life, including physical, psychological, spiritual and social aspects [7,8,9]. As such, it is not surprising that HF is associated with poor HRQOL, given that patients with HF present with a cluster of incapacitating symptoms ranging from physical signs and symptoms (dyspnea, orthopnea, bendopnea, nocturnal cough, fatigue, exercise intolerance, palpitations, dizziness, bloating, peripheral edema, ascites, pain, cachexia) to psychological, emotional and mental manifestations (confusion, depression, anxiety, sleep disorders, cognitive impairment) [1,10,11,12]. These symptoms, either individually or collectively and oftentimes in conjunction with adverse effects from HF treatment per se, interfere with activities of daily living, limit functional performance and disrupt multiple domains of life, ultimately impairing patient’s well-being and HRQOL and rendering the patient dependent on caregivers, while leading to social isolation, anxiety and depression [13,14,15,16,17,18,19]. Of note, HRQOL seems to be impaired even in the early asymptomatic phases of HF and continues to deteriorate as HF gradually becomes more overt [20]. More importantly, low levels of HRQOL in patients with HF portend a poor prognosis, since they have been associated with unfavorable outcomes in terms of morbidity and mortality [21,22,23]. 

**Figure 1:**
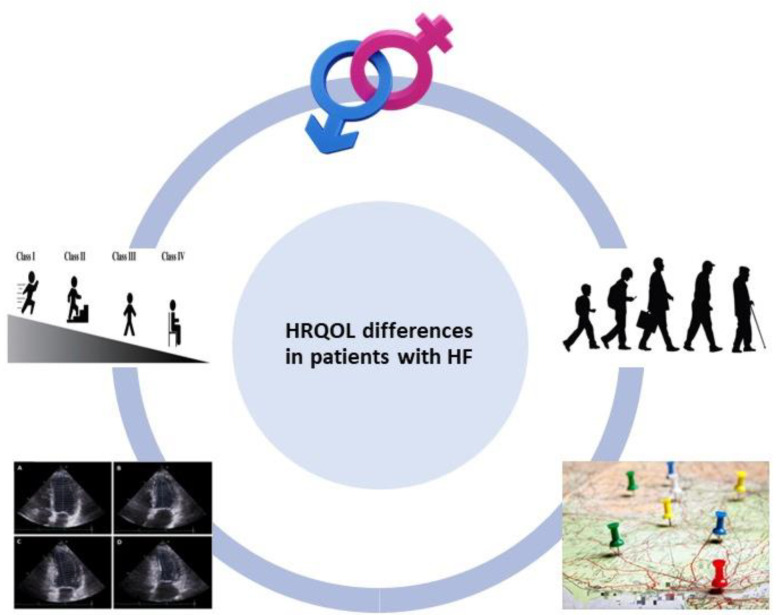
Differences in health-related quality of life (HRQOL) among patients with heart failure (HF) based on sex, age, New York Heart Association (NYHA) functional class, ejection fraction and geographic location or ethnicity. For the construction of Figure 1, images from references [28,29] have been used.

It is remarkable that patients with HF display lower levels of HRQOL compared not only to the general healthy population but also to patients suffering from other chronic diseases [24,25,26,27]. However, even among patients with HF, it has been consistently reported that there are existing differences in HRQOL. Accordingly, the aim of this review article is to highlight underlying differences in HRQOL among patients with HF and provide a comprehensive overview of currently existing evidence with regard to observed disparities. As depicted in Figure 1, the fundamental differences in HRQOL among patients with HF are analyzed based on sex, age, New York Heart Association (NYHA) functional class, EF and geographic location or ethnicity. 

## 2. Assessment of HRQOL

In order to assess HRQOL in patients with HF, patient-reported outcome measures have been increasingly utilized, which are self-reports stemming directly from patients with no intervention or interpretation by healthcare providers, most commonly in the form of self-administered questionnaires or interviews. These questionnaires serve as instruments of measuring patients’ experiences and perspectives with regard to their disease. HRQOL questionnaires are intended to assess the impact of the disease on the daily living of patients, in the best possible and most accurate way, and identify how patients perceive their condition, experience possible limitations regarding their engagement in activities and cope with the challenges of their disease. Apart from that, HRQOL assessment tools have been increasingly included as endpoints in many clinical trials and have been consistently used as potential instruments to support the choice of a certain therapeutic strategy, evaluate the effectiveness of a treatment or even its side effects and guide future patient care [30,31,32,33].

As HF management is becoming more patient-centered, there is an ever-increasing interest in the use of patient-reported outcomes as surrogate endpoints in clinical trials and the utilization and implementation of HRQOL measures in clinical decision-making and therapeutic intervention. HRQOL measures inform therapeutic choices and disease management practices, while they also carry readily available prognostic information, thereby being useful for HF surveillance and prognostication [33,34,35,36,37]. Accordingly, it comes as no surprise that optimizing HRQOL, as a patient-centered outcome, has been acknowledged as one of the main goals in the management and the shared decision-making process for patients with HF [38], while strategies to enhance HRQOL in these patients have been strongly supported by the guidelines of major scientific societies of cardiology [1,2]. The importance of maintaining or improving HRQOL is highlighted by the fact that maintaining a good HRQOL has long been conceived by many patients with HF as at least as equally important as survival [39]. In fact, many patients with HF prefer better HRQOL to longevity and state that they would be willing to trade longer life expectancy for better HRQOL [40].

HRQOL assessment tools can be either generic, which provide an overall evaluation of the health status of different populations, or disease-specific, which assess specific aspects of HRQOL pertinent to the particular disease, in this case HF [32]. Examples of generic HRQOL assessment tools are the following: Medical Outcomes Study 36-Item Short Form Health Survey questionnaire (SF-36), or its more brief version, the 12-Item Short-Form Health Survey questionnaire (SF-12); EuroQol five-dimensional questionnaire (EQ-5D) which exists in the forms of EQ-5D 3-Level (EQ-5D-3L), EQ-5D 5-Level (EQ-5D-5L) and EQ Visual Analogue Scale (EQ-VAS); and Patient-Reported Outcomes Measurement Information System (PROMIS) [24,41,42,43,44]. On the other hand, the most widely used HF-specific tools for the assessment of HRQOL in patients with HF are the Minnesota Living with Heart Failure Questionnaire (MLHFQ) [45] and the Kansas City Cardiomyopathy Questionnaire (KCCQ), along with its brief version, the KCCQ-12, which consists of 12 instead of the original 23 items [46]. 

MLHFQ is a 21-item HF-specific questionnaire. Of the 21 items, 8 correspond to the physical dimension and 5 capture the emotional dimension, whereas the remaining 8 items are merely utilized for the calculation of the total MLHFQ score. All items are rated on a 6-point Likert scale (0–5), with 0 denoting no effect of HF on HRQOL and 5 indicating the highest degree of impact. The scoring of the physical dimension ranges from 0 to 40, while the scoring of the emotional dimension spans from 0 to 25. The combination of scores from all 21 items yields a total score, which ranges from 0 to 105. Owing to the fact that 0 on the Likert scale represents no impact of HF on HRQOL, lower total MLHFQ scores reflect better HRQOL and higher scores indicate worse HRQOL [45,47,48]. On the other hand, KCCQ is composed of 23 items incorporated into 15 questions, which cover the following seven domains: physical limitations, symptom stability, symptom frequency, symptom burden, self-efficacy, social limitations and quality of life. Each item is scored on a Likert scale with five to seven response options. Scores from individual domains are selectively merged to form three summary scores (symptom summary score, clinical summary score and overall summary score). The score range spans from 0 to 100, with lower scores depicting poorer HRQOL [46,49,50,51].

The abundance of both generic and HF-specific HRQOL measures used in clinical research and clinical trials may in part account for disparities in HRQOL observed among patients with HF. Oftentimes, theoretical and methodological issues may arise owing to the lack of conceptual clarity and inconsistent use of HRQOL measures which may hinder reliable replicability, preclude comparison, yield counterintuitive results and cause ambiguity [7,8,52,53]. Besides that, many observed disparities in HRQOL among patients with HF could be attributed to discrepancies in demographic variables, socioeconomic status, social support, healthcare resources or accessibility of healthcare systems. Diverse spiritual attitudes and cultural responses to symptom burden, disability and disease in general could also account for differences in HRQOL. Furthermore, heterogeneity in body structure and function, inherent factors related to idiosyncratic features and environmental or even political factors may just as well play a contributing role [8,54,55,56]. 

## 3. Sex-Related Differences in HRQOL

Several studies point towards existing differences in HRQOL among patients with HF based on sex. In particular, it has been consistently observed that women with HF tend to have lower HRQOL levels compared to men. In a recent systematic review and meta-analysis by Moradi et al., female patients with HF had a lower HRQOL than their male counterparts, as evidenced by a higher pooled mean total MLHFQ score in women (45.6) compared to men (40.7). For purposes of clarity, it should be emphasized that in the MLHFQ questionnaire, higher scores correspond to poorer HRQOL [24]. 

Similar sex-based differences in HRQOL have also been observed in an analysis of two large randomized controlled trials including 15,415 patients with HFrEF from 55 countries, namely the prospective comparison of angiotensin receptor neprilysin inhibitor with an angiotensin converting enzyme-inhibitor to determine impact on global mortality and morbidity in heart failure (PARADIGM-HF) and Aliskiren trial to minimize outcomes in patients with heart failure (ATMOSPHERE). HRQOL was measured in 13,061 patients by means of the KCCQ score and it was found that women had worse median KCCQ scores. Actually, women had lower scores in all individual KCCQ domains (physical limitation, symptom frequency, symptom burden, self-efficacy, quality of life, social limitation) except for one (symptom stability). The largest difference was observed in the domain of physical limitation [57].

Identical results were reported in another study which analyzed 1649 patients with HFrEF from 11 European countries who had participated in the systems biology study to tailored treatment in chronic heart failure (BIOSTAT-CHF) trial. The reported HRQOL at baseline was significantly worse in women than in men, when assessed with the use of both disease-specific and generic tools, namely KCCQ and EQ-5D questionnaires, respectively. The KCCQ overall score was significantly lower in women (43.8) than in men (53.1). Lower scores were observed in six out of seven KCCQ individual domains, with symptom stability being the only domain that did not reach statistical significance. The most prominent sex differences were noted in the KCCQ domains of physical and social limitation. Notably, fatigue was the most prominent limiting symptom in women, who experienced more severe physical limitations than men when the intensity of physical activities increased. Additionally, HF was reported to pose a greater impact on all social activities in women compared to men. With regard to the EQ-5D questionnaire, women scored worse in all five dimensions (mobility, self-care, usual activities, pain/discomfort, anxiety/depression), indicating a significantly higher burden of both physical and psychological limitations [58].

Likewise, in an analysis of the Spanish multicenter study Quality of life and heart failure in Spain: Current situation (VIDA-IC), HRQOL was assessed in 1028 patients with HFrEF by means of two questionnaires (KCCQ and EQ-5D). It was found that women experienced worse HRQOL than men, as evidenced by lower overall summary score in the KCCQ. Lower scores were observed in the domains of physical limitation, symptom frequency, symptom burden, quality of life and social limitation. Regarding EQ-5D, women displayed lower scores in all individual dimensions [59]. 

Of particular interest is a retrospective analysis of a bi-national study (KaRen-Karolinska Rennes) that assessed the impact of sex on HRQOL in an unselective cohort of patients suffering exclusively from HFpEF, which is known to be characterized by female predominance. A total of 378 patients with HFpEF were included from Sweden and France, 57% of whom were women. HRQOL was assessed with the use of a generic (EQ-5D-3L) and a disease-specific (MLHFQ) tool. Based on EQ-5D-3L, women reported worse HRQOL in all five dimensions of the descriptive part of the questionnaire, but especially in the dimensions of mobility, usual activities and anxiety/depression. In the second part of the questionnaire, the EQ-VAS, women self-rated their global health with lower values than men, denoting worse HRQOL. This discrepancy in HRQOL between men and women persisted even after adjusting for age and HF severity. On the other hand, when MLHFQ was used, no significant difference in HRQOL was observed between the two sexes. Furthermore, irrespective of the assessment tool used, HRQOL was correlated with HF severity in both sexes, but the correlation was stronger in men. Even more so, poorer HRQOL was significantly associated with adverse outcomes only in men [60]. Similar conclusions were drawn from another study of patients with HFpEF, which was actually a pooled secondary analysis of two HFpEF trials: phosphodiesterase-5 inhibition to improve clinical status and exercise capacity in heart failure with preserved ejection fraction (RELAX) and nitrate’s effect on activity tolerance in heart failure with preserved ejection fraction (NEAT-HFpEF). HRQOL measured by MLHFQ did not differ between men and women, while exercise capacity measured by 6-min walk test (6MWT) distance was associated with HRQOL only in men but not in women [61]. On the contrary, in a post hoc analysis of the treatment of preserved cardiac function heart failure with an aldosterone antagonist (TOPCAT) trial, which included patients with HFpEF enrolled exclusively from the Americas cohort, it was noted that women displayed lower HRQOL, as indicated by a lower KCCQ overall score, while they also exhibited higher levels of depression, reflected by a higher score in Patient Health Questionnaire-9 (PHQ-9) [62]. Consistent with these findings were the results of another pooled analysis, which included a cohort of 8468 patients with HFpEF from three large clinical trials: Candesartan in heart failure: Assessment of reduction in mortality and morbidity-Preserved (CHARM-Preserved), irbesartan in heart failure with preserved ejection fraction (I-Preserve) and TOPCAT-Americas. In this analysis, women had poorer HRQOL than men, which was accompanied by worse NYHA functional class and higher burden of symptoms and signs of HF [63].

The greater burden of HF on women’s HRQOL has also been corroborated by a recent real-world cross-sectional study which included 804 patients from five European countries (France, Spain, Italy, Germany and United Kingdom) presenting with an LVEF ≤ 60%, thus including all HF phenotypes, namely HFrEF, HFpEF and HFmrEF. Women exhibited consistently poorer HRQOL than men, as indicated by both significantly higher (worse) MLHFQ scores and significantly lower scores in all scales of the EQ-5D-5L questionnaire. Lower HRQOL in women was evident across both physical and emotional domains. Symptoms more commonly experienced by women than men were fatigue, edema and palpitations, with fatigue and edema being reported by women as the most troublesome ones. Moreover, women experienced depression and anxiety more often than men. Regarding the impact of HF on the activities of daily living, both sexes reported similar levels of disruption. No significant sex-related differences were noted in the overall work productivity; however, women reported a higher percentage of activity impairment due to HF compared to men [64]. Similar findings were reported by a 1-year longitudinal prospective study from China, which included 154 patients with HF across all EF ranges. In this study, HRQOL was assessed by means of two tools (KCCQ and EQ-5D) at various timepoints (1, 6 and 12 months) and it was found to be significantly lower in women than in men at each follow-up, although paradoxically at baseline HRQOL was similar in both sexes [65].

Sex-related differences have also been observed among patients with advanced HF. A secondary analysis of the palliative care in heart failure (PAL-HF) trial concluded that advanced HF poses a heavier symptom burden on women, who consequently experience worse HRQOL, as evidenced by a lower median KCCQ score compared to men. Moreover, depression was more prevalent in women, who were more often widowed and more likely to be inert by spending most of their time in bed. The same study assessed the impact of palliative care intervention on HRQOL of men and women after 24 weeks of a multidisciplinary palliative approach. It was found that at 24 weeks men who received palliative care had higher median KCCQ score than those who received usual medical care, whereas such a significant benefit of palliative care intervention was not observed in women, whose HRQOL consistently remained lower than that of men throughout the study period [66].

Disparities in HRQOL between the two sexes are also evident in the acute HF setting. The acute study of clinical effectiveness of nesiritide in decompensated heart failure (ASCEND-HF) trial included a global cohort of 7141 patients hospitalized due to acute HF. Their HRQOL was assessed with the use of EQ-5D questionnaire at different timepoints during and after their hospitalization (at baseline, upon discharge and at 30 days). In all instances, women were found to have greater impairment in HRQOL than men on serial assessments extending throughout their acute HF course, from hospital admission through day 30. These sex differences persisted even after adjusting for other demographic and clinical parameters [67].

It is unclear why women display lower levels of HRQOL compared to men. This could be partially explained by the fact that women experience more symptoms and signs of HF than men; yet, one would expect that the greater symptom burden would be accompanied by worse physiological markers of HF severity (EF, N-terminal pro-brain natriuretic peptide (NT-proBNP), prior HF hospitalization), which is, however, not the case [57]. One could also assume that worse HRQOL in women could merely reflect the underlying preponderance of women in the subgroup of patients with HFpEF; yet, this explanation is not sufficient per se, since worse HRQOL has also been observed in women with HFrEF, a subtype of HF characterized by male predominance [68,69,70,71]. In the latter subtype of HFrEF, a feasible explanation of worse HRQOL in women could be related to the fact that women have been persistently undertreated in terms of both HF-specific pharmacologic agents and devices. In addition to that, women are less likely to be referred to cardiac rehabilitation programs [57,68,72,73]. Higher levels of anxiety and depression in women could also be responsible for the observed sex-based differences in HRQOL [57,61,68,73]. Other factors that may be contributing to the lower levels of HRQOL in women could be related to psychosocial, socioeconomic or educational aspects, restricted access to healthcare systems, limited caregiver support, higher incidence of solitary living, lower rates of seeking help, more difficulties with adapting to the disease and the underrepresentation of women in clinical trials. Nevertheless, HF seems to exert a more unfavorable impact on the lives of women, which is reflected by more severe signs and symptoms of HF, higher physical and psychological burden and worse HRQOL [24,57,59,61,68,72,73]. Table 1 summarizes studies which have evaluated sex-related differences in HRQOL among patients with HF. 

## 4. Age-Related Differences in HRQOL

A vast majority of studies point towards the fact that disparities in HRQOL exist among different age groups of patients with HF. In general, HRQOL seems to be better in older patients with HF compared to younger ones. Wong et al. analyzed data from a combined cohort of patients with HFrEF and HFpEF who participated in the Candesartan in heart failure: Assessment of reduction in mortality and morbidity (CHARM) program. The analysis was conducted by categorizing patients into five age groups: 20–39, 40–49, 50–59, 60–69 and ≥70 years old. HRQOL was assessed in patients from the United States of America (USA) and Canada by using MLHFQ. It was found that the worst HRQOL was reported by the youngest patients, despite the fact that they had a more favorable NYHA class profile. With increasing age, HRQOL steadily improved. The paradox that younger patients with HF experience worse HRQOL may be attributed to the fact that younger patients lead by default a more active and demanding life and face greater challenges in coping with various aspects of everyday living, such as family, work, child raising and social commitments. Within this framework, HF symptoms and the subsequent limitation in functional capacity may exert a greater impact on younger patients, who consequently perceive their HRQOL as being worse. In keeping with the above, non-adherence with medication, dietary restriction and healthy lifestyle measures, which was found to be higher in younger patients, may have also contributed to the worse levels of HRQOL in this age group [74]. 

A recent study analyzed data of patients with HFpEF who participated in three multicenter clinical trials: RELAX, NEAT-HFpEF and inorganic nitrite delivery to improve exercise capacity in heart failure with preserved ejection fraction (INDIE-HFpEF). Two HF-specific instruments were used to assess HRQOL: MLHFQ in the RELAX trial and KCCQ in the other two trials. Regardless of the instrument used, the worst HRQOL was observed in the youngest patients and especially among those with comorbidities, such as obesity and diabetes mellitus [75]. Similar findings were reported by another study which utilized data from three large HFpEF trials, namely CHARM-preserved, I-PRESERVE and TOPCAT. Again, HRQOL was worse in younger patients, irrespective of the tool used (MLHFQ or KCCQ). This association between worse HRQOL and young age remained significant even after adjusting for sex, body mass index, diabetes and history of atrial fibrillation [76]. 

On the contrary, the Asian sudden cardiac death in heart failure (ASIAN-HF) trial, which investigated age-related differences in 1203 patients with HFpEF from 11 Asian countries, found that younger patients had better HRQOL (based on KCCQ scores) compared to their older counterparts [77]. Contradictory results were also reported by a study which analyzed data drawn from the Swedish Heart Failure Registry. HRQOL was measured by means of EQ-VAS. The study included 23,533 patients, with median age of 74 years, who were stratified by age into the following 5-year-interval categories: ≤60, 61–65, 66–70, 71–75, 76–80, 81–85 and >85 years old. It was found that patients in the oldest group displayed worse HRQOL (expressed as lower median EQ-VAS scores) than those in the youngest one. However, an interesting trend was observed; HRQOL initially showed improvement from the youngest age group up to the age category of 66–70 and started deteriorating thereafter. Compared to other studies which have reported worse HRQOL in younger ages, this particular study used a different HRQOL tool and, more importantly, it recruited generally older patients and stratified them into narrower age groups. This may have partially accounted for the observed discrepancy [78]. 

Nevertheless, an Australian-based study, which investigated HRQOL among a very elderly cohort of community-dwelling patients with HF (mean age 80.93 years), found that younger patients had more impaired HRQOL in all MLHFQ subscales (physical, emotional and overall) [79]. A negative correlation between HRQOL and age has also been observed in elderly patients who had been recently hospitalized due to decompensated HF. HRQOL was assessed within a month after hospital discharge by means of MLHFQ. This observational study included 1911 patients with a mean age of 79 years and showed that older patients had higher levels of HRQOL. When the entire cohort of patients was dichotomized into two groups (≤80 and >80 years old), it was noted that determinants of HRQOL differed between the two groups. For patients ≤80 years old, predictors of worse HRQOL were younger age, lower levels of hemoglobin and presenting symptoms of peripheral edema, exertional dyspnea and fatigue at the time of HF hospitalization. For patients >80 years old, chronic kidney disease and presenting symptoms of exertional dyspnea and peripheral edema predicted worse HRQOL, whereas living alone was surprisingly associated with better HRQOL. Lower levels of HRQOL could independently predict HF readmissions only in patients aged >80 years, but not in those aged ≤80 years [80].

It is noteworthy that, despite the fact that in most clinical trials older patients with HF display higher baseline levels of HRQOL compared to younger ones, over time they run a greater risk of HRQOL deterioration if they suffer an acute decline in their functional status. This notion was supported by a multicenter prospective cohort study which assessed 484 patients with HF longitudinally (at baseline and 6 ± 2 weeks later). Analysis included comparison of older (≥65 years old) versus younger (<65 years old) patients in terms of HRQOL and functional capacity at different timepoints. KCCQ was used to measure HRQOL, whereas NYHA classification and 6MWT were deployed for the evaluation of functional status. At baseline, older patients had better HRQOL in spite of worse functional status. At follow-up, older patients, whose functional capacity was aggravated, experienced a statistically significant deterioration in their HRQOL. On the contrary, no significant change in HRQOL was noted in younger patients who had experienced a decline in functional status over the short term [81]. 

Age-related differences have also been observed in patients with advanced HF who receive more sophisticated HF treatment. A study examined HRQOL in a population of 287 patients with advanced HF and symptoms refractory to optimal medical and device therapy, who were receiving outpatient treatment with intermittent low-dose intravenous inotropes. HRQOL was assessed at baseline and after one year of treatment by using MLHFQ. Younger patients had significantly worse HRQOL at baseline compared to older ones. Nonetheless, after one year of treatment, younger patients were more likely to show improvement in their HRQOL, which, however, did not translate into better long-term clinical outcomes in terms of a survival benefit [82].

Considering the fact that elderly patients generally have more physical limitations and worse prognosis due to their age per se, it is intriguing that most studies report that older patients with HF display a better HRQOL profile. Driven by this paradox, Moser et al. aimed to explore the underlying reasons of this counterintuitive phenomenon. They included 603 patients with HF and stratified them into four age groups: ≤53, 54–62, 63–70 and ≥71 years old. HRQOL was evaluated with the use of MLHFQ. They confirmed the finding that older patients had better HRQOL, as well as lower levels of psychological distress. Anxiety, depression and functional capacity were independent predictors of HRQOL in all age groups. In a qualitative analysis, older patients mentioned that their HRQOL surpassed their personal expectations, considering their age, while they also tried to make up for their lost ability to perform certain tasks by adopting alternative activities. On the other hand, younger patients with HF found it difficult to accept the changes imposed to their lives by their underlying cardiac condition and seemed to have a denial regarding the loss of their active roles and their inability to perform particular activities due to physical constraints. Their feeling of loss, coupled with their unwillingness to change personal expectations and adapt their lifestyle according to their HF status, had a very negative impact on their psychological outlook, since they felt devastated about not being able to lead vigorous, functional and productive lives anymore, at least not to the same extent as they used to in the past. The authors concluded that better HRQOL in older patients with HF may be ascribed to their better psychosocial status and to their altered conceptions about what constitutes a good HRQOL, which in turn lead to lower expectations in life with advancing age [83]. A summary of studies reporting age-related differences in HRQOL among patients with HF is presented in Table 2.

## 5. Differences in HRQOL Based on NYHA Functional Class

The impact of NYHA functional class on HRQOL has long been appreciated. In the early 2000s, Juenger et al. assessed HRQOL in 205 patients with congestive HF by using SF-36, which is a generic tool consisting of eight subscales that cover the domains of physical functioning, role functioningphysical, bodily pain, general health perceptions, vitality, social functioning, role functioning—emotional and mental health. With worsening NYHA class, a gradual decline in HRQOL scores was observed. The domains of physical functioning, role functioning—physical and role functioning—emotional were predominantly affected. Notably, in NYHA class I, subscales that were mainly compromised were those related to physical functioning, whereas, in NYHA classes II and III, all domains gradually became severely afflicted, including those of emotional wellbeing and functioning [25]. Likewise, a Japanese study, which was conducted almost 20 years ago, investigated the relationship between HRQOL and different levels of HF severity, expressed as NYHA class. HRQOL was measured by means of SF-36. The study population comprised 125 ambulatory patients with HFrEF. It was noted that HRQOL decreased as NYHA functional class increased [84]. Similar findings were reported by a subsequent Spanish study, which utilized SF-36 and MLHFQ for the evaluation of HRQOL in 544 patients with clinically stable HF. Again, patients in NYHA class III–IV had significantly poorer HRQOL, with physical domains being more impaired than emotional ones [85].

Nesbitt et al. explored HRQOL in a United States (US) population of 612 patients with HF who were living exclusively in rural areas of three different states. MLHFQ was used for the measurement of HRQOL and it was found that patients with worse functional capacity, designated by higher NYHA class, displayed more impaired HRQOL [86]. Another study investigated the correlation between NYHA class and HRQOL in 152 patients with HF. HRQOL was assessed by consecutively administering three questionnaires, two HF-specific (MLHFQ, KCCQ) and one generic (EQ-5D-3L), to each patient in a set order. There was good correlation among mean HRQOL scores derived from all three HRQOL instruments, with the strongest correlation being observed between MLHFQ total score and KCCQ overall summary score. On univariable analysis, NYHA class turned out to be the only variable that showed significant correlation (*p* < 0.01) with each of the three HRQOL instruments (MLHFQ, r = 0.59; KCCQ, r = −0.61; EQ-5D-3L, r = −0.44). The association was strongest with the HF-specific instruments, KCCQ and MLHFQ. On multivariable linear regression analysis, the significant association between NYHA class and HRQOL scores of each of the three questionnaires persisted (all *p* < 0.005). Despite the significant association between NYHA class and HRQOL, the authors underlined the fact that HRQOL scores were highly variable between individual patients within each NYHA class, suggesting that NYHA functional class cannot capture all aspects of HRQOL at an individual level [87].

In a recent Canadian study, which included 270 patients with all HF subtypes (53.2% HFrEF, 30.3% HFpEF and 16.5% HFmrEF), HRQOL was estimated using the KCCQ-12 questionnaire, while factors predicting HRQOL were also evaluated. In multivariate analysis, advanced NYHA class (III or IV) emerged as one of the few independent determinants of poorer HRQOL [88]. In addition, the SENECOR (intervention by a cardiologist and geriatrician in elderly patients after admission due to heart failure) study examined HRQOL in a cohort of 141 elderly patients (≥75 years old) with HF who had been recently hospitalized. HRQOL was measured with the use of KCCQ-12. It was found that patients who were in higher NYHA classes, and thus had more functional limitations, tended to have worse HRQOL. Worse HRQOL was also associated with greater frailty and worse performance in the activities of daily living, as measured by the Barthel index [89]. By the same token, in a cross-sectional study of 175 patients with HF aged ≥60, it was noted that HRQOL, assessed through MLHFQ, was more impaired in patients who were in NYHA classes III and IV than in those who were in NYHA classes I and II. By using a series of hierarchical regression analyses, the investigators demonstrated that NYHA class exerted an indirect impact on HRQOL through depression, which acted as a mediator. They concluded that efforts should be made by healthcare professionals to improve NYHA functional class substantially in order to manage comorbid depression effectively and thus enhance HRQOL in older adults with HF [90]. Additionally, higher NYHA class and depression have been shown to be significantly associated with worse HRQOL in older patients with advanced HF who are either candidates for heart transplantation or have been scheduled for long-term mechanical circulatory support as destination therapy [91]. 

## 6. Differences in HRQOL Based on Ejection Fraction

Several studies have focused on the differences in HRQOL that may exist among patients with different HF subtypes according to their EF. Chen et al. examined HRQOL in 841 hospitalized patients with HF. Patients were stratified into three groups (HFrEF, HFmrEF and HFpEF) based on their EF value (<40%, 40–49% and ≥50%, respectively) and their HRQOL was assessed by using MLHFQ. MLHFQ scores, including total scores as well as physical and emotional subscale scores, differed significantly among the three groups. Patients with HFrEF displayed higher (worse) MLHFQ scores in all domains, consistent with poorer HRQOL, compared to patients with HFpEF and HFmrEF. When analyzing the interaction between HF subtype and MLHFQ score, it was noted that the total MLHFQ score and the physical subscale score were significantly affected by the HF subtype, whereas no such association was observed for the emotional subscale score. Of note, as the overall and physical scores increased (worsened), the observed statistical significance became even more pronounced. When comparing patients with higher MLHFQ scores (>39), hence lower HRQOL, it was observed that patients with HFrEF had significantly worse clinical outcomes at one year than patients with HFpEF and HFmrEF. On the contrary, among patients with the best HRQOL, indicated by the lowest MLHFQ scores (<28), it was found that patients with HFpEF had lower rates of adverse outcomes compared to patients with HFmrEF [92]. In addition, in the global congestive heart failure (G-CHF) study, a multinational cohort study, HRQOL was measured by means of KCCQ-12 in more than 23,000 patients with HF. For the purposes of the analysis, patients with HFmrEF and patients with HFpEF were merged into one group (EF ≥ 40%), whereas patients with HFrEF formed the second group (EF < 40%). This was carried out in order to allow for comparisons between groups of relatively equal size. The group of patients with EF ≥ 40% had better HRQOL, as evidenced by higher KCCQ-12 summary score, compared to the group of patients with HFrEF. When looking at the relationship between clinical outcomes and EF category at different HRQOL levels, it was observed that, among patients with better HRQOL (KCCQ-12 summary score ≥50), patients with EF < 40% had higher mortality rates than patients with EF ≥ 40%. By contrast, among patients with worse HRQOL (KCCQ-12 summary score ≤49), mortality rates were equally poor in patients with HFrEF and patients with EF ≥ 40% [93]. 

As opposed to the aforementioned studies, other studies have reported that patients with HFpEF, and not patients with HFrEF, exhibit poorer HRQOL. Indeed, a study retrospectively analyzed data from patients by stratifying them into different EF ranges: 1058 patients with HFrEF, 185 patients with HFmrEF and 162 patients with HFpEF. By using MLHFQ for HRQOL assessment, they found that HRQOL did not differ between patients with HFrEF and HFmrEF, whereas it was worse in patients with HFpEF [94]. Likewise, in a very recent large study analyzing data from the Swedish Heart Failure Registry, it was observed that patients with HFmrEF had the best HRQOL (measured by EQ-VAS), whereas patients with HFpEF displayed the worst HRQOL, followed by patients with HFrEF [78]. Another study investigated HRQOL in 3499 patients with HF, who were pooled from the patient cohort of the BIOSTAT-CHF trial. Patients belonging to all HF subtypes were included: 2309 patients had HFrEF, 634 patients had HFmrEF and 556 patients had HFpEF. HRQOL was assessed by using one HF-specific (KCCQ) and one generic (EQ-5D) tool. With regard to the KCCQ instrument, overall HRQOL was lower in patients with HFpEF than patients with HFrEF and HFmrEF. Specifically, patients with HFpEF exhibited more impaired HRQOL in the domains of physical limitations, social limitations, symptom frequency and symptom burden. Furthermore, the presence of comorbidities was associated with poorer HRQOL, with the association being more pronounced in patients with HFrEF. The strongest association with poorer HRQOL in all HF subtypes was consistently noted for chronic obstructive pulmonary disease. As expected, multiple comorbidities were more prevalent in patients with HFpEF. When using EQ-5D, the observed differences in HRQOL and its association with comorbidities among HF groups were attenuated [95]. In addition, Bekfani et al. conducted a small-scale study in clinically stable outpatients with HFrEF (EF < 40%) and HFpEF (EF ≥ 50%) and estimated their HRQOL by using SF-36 and EQ-VAS. They found that patients with HFpEF had worse HRQOL, especially in the domains of mental health and vitality in the SF-36 questionnaire, compared to patients with HFrEF. Moreover, patients with HFpEF demonstrated higher levels of depression and anxiety [96]. Along the same lines, in a study including exclusively hospitalized patients with acute decompensated HF, HRQOL was reported to be more diminished in patients with HFpEF (EF ≥ 45%) compared to patients with HFrEF (EF < 45%), even after adjustment for demographic and clinical variables. In this case, HRQOL was estimated with the use of three tools (KCCQ, SF-12 and EQ-5D-5L) and the finding of worse HRQOL in HFpEF patients was consistently evident, irrespective of the HRQOL tool used. Furthermore, HFpEF patients presented with more depressive symptoms, which were strongly predictive of poorer HRQOL [97]. 

On the other hand, there are studies reporting no significant variations in HRQOL among patients with HF who belong to different EF groups. In 2007, the CHARM HRQOL study investigated HRQOL with the use of MLHFQ in 2709 patients with symptomatic chronic HF by stratifying them into two groups based on their EF: HFrEF (≤40%) and HFpEF (>40%). At that time, the notion of HFmrEF had not been introduced yet. When comparing HRQOL between the two groups, the authors did not find any considerable differences either in the total MLHFQ score or in the emotional dimension score, whereas the physical dimension score was slightly worse in patients with HFpEF. They concluded that both patients with HFrEF and HFpEF had equally impaired HRQOL [98]. A subsequent study by Hoekstra et al. corroborated the above finding by utilizing three different assessment tools: Cantril’s Ladder of Life for the assessment of global well-being, RAND-36 for the assessment of general health status and MLHFQ for the assessment of disease-specific HRQOL. Regardless of the tool used, patients with HFpEF (EF ≥ 40%) had similarly affected HRQOL as patients with HFrEF (EF < 40%) [99]. Similar findings were recorded in the Alberta heart failure aetiology and analysis team (HEART) study, which investigated longitudinal changes in HRQOL in 360 patients with HF. Both at baseline and at 12-month follow-up, patients with HFpEF (EF ≥ 45%) presented with numerically lower KCCQ scores, depicting worse HRQOL, compared to patients with HFrEF (EF < 45%). However, the observed difference did not reach statistical significance. Furthermore, longitudinal changes in the KCCQ score over the period of 12 months could not be predicted by HF subtype per se; yet, a decline in the KCCQ score was found to be more strongly associated with adverse outcomes in patients with HFpEF than in patients with HFrEF [100]. Additionally, in a study which included 622 patients with HF covering the whole spectrum of EF, it was found that HRQOL, assessed by MLHFQ and SF-12, was almost equally impaired in patients with HFrEF, HFmrEF and HFpEF [101]. Similar results have been reproduced by small-scale studies, which have reported that HRQOL did not differ between patients with HFrEF and HFpEF when utilizing either HF-specific or generic HRQOL assessment tools [102,103]. 

A recent study compared HRQOL between patients from two major clinical trials of sacubitril/valsartan; that is, 4735 patients with HFpEF (EF ≥ 45%) from the prospective comparison of angiotensin receptor neprilysin inhibitor with angiotensin receptor blocker global outcomes in HFpEF (PARAGON-HF) trial and 6887 patients with HFrEF (EF ≤ 40%) from the PARADIGM-HF trial. It was noted that both patient populations shared the same demographic and clinical factors that adversely affected HRQOL, with the strongest ones being NYHA class, female gender, peripheral edema, dyspnea at rest and on exertion, paroxysmal nocturnal dyspnea, angina, higher body mass index, chronic obstructive pulmonary disease, geographic region and log NT-proBNP. In unadjusted models, patients with HFpEF demonstrated impaired HRQOL (assessed by KCCQ) to a greater extent than patients with HFrEF. Indeed, patients with HFpEF exhibited lower mean scores in nearly all KCCQ domains (except for the domains of symptom stability, quality of life and social limitation) and in almost all physical and social activities (excluding intimate or sexual relationships). However, after adjusting for the aforementioned clinical and demographic factors, the previously observed differences in the KCCQ overall summary scores lost their statistical significance, thus signaling the fact that the overall HRQOL was similar in patients with HFrEF and HFpEF in the adjusted models. Notwithstanding, patients with HFpEF continued to display significantly lower KCCQ clinical summary scores than patients with HFrEF, albeit with a diminished magnitude of statistically significant difference [104]. 

HRQOL has also been evaluated in patients with recovered or improved EF (HFimpEF). This term is used for a distinct type of HF characterized by a baseline EF value ≤40%, which increases to an EF value >40% at follow-up, provided that the absolute increase in EF from baseline is ≥10% [2,3]. A Spanish study analyzed data from a registry of 1040 outpatients with HFrEF who were followed for one year. At 1-year follow-up, 34.7% of these patients fell into the category of HFimpEF. HRQOL was evaluated with the use of MLHFQ. Surprisingly, at baseline, patients with HFimpEF had higher MLHFQ score (worse HRQOL) than patients who did not fulfill the criteria for HFimpEF. However, at 1 year, there was no statistically significant difference in HRQOL between these two groups. This was attributed to the fact that patients with HFimpEF demonstrated a significantly higher improvement in HRQOL over the 1-year period than those who remained in the category of HFrEF without improved EF. The improvement in HRQOL was primarily ascribed to the reduction in the number of HF-related hospitalizations over the previous year and to the improvement in NYHA class. Nevertheless, it was noted that, in patients with HFimpEF, this longitudinal improvement in HRQOL from baseline was not associated with clinical outcomes, whereas their MLHFQ score at 1 year was strongly related to outcomes and served as an independent prognostic factor [105]. Another prospective cohort study examined changes in HRQOL in 319 patients with HF (212 with HFrEF and 107 with HFpEF) over a period of 1 year and found that patients with HFrEF whose EF had recovered to a value of 50% or more displayed significant improvement in HRQOL. The authors further quantified the correlation between change in HRQOL and EF and reported a mean increase of 4.8 points in the KCCQ-12 overall summary score with every 10% increase in EF. Contrary to patients with recovered EF, patients who remained in the HFrEF category showed a much smaller improvement, whereas patients with HFpEF had no significant change in HRQOL [106]. Likewise, in a large cohort of outpatients with HFrEF, which was pooled from the Change the Management of Patients with Heart Failure (CHAMP-HF) registry, increases in EF of ≥10% over time were associated with improved HRQOL as well as with a decreased risk for future clinical outcomes [107]. In an earlier study, patients with HF and recovered EF were found to have significantly higher overall HRQOL and lower dyspnea burden compared to patients with HFpEF. However, definitions used in this study differed from contemporary ones, since HFrEF was defined as persistent EF < 50% and HFpEF as EF ≥ 50%, while patients with recovered EF were deemed as those with prior HFrEF (EF < 50%) whose EF had improved to ≥50% [108].

Based on the above findings, it becomes evident that conflicting results have been reported regarding HRQOL in different HF subpopulations according to EF. This can be partially attributed to the varying cut-offs used in different clinical trials for the stratification of patients into a certain HF subtype (HFpEF, HFrEF, HFmrEF and HFimpEF), coupled with the fact that the universal definition and classification of HF had not been introduced until recently, thus creating a new scientific landscape with a revised classification scheme to which clinical research and HF trials have not adapted yet [3]. The observed discrepancies may also be due to the different HRQOL assessment tools deployed, the heterogeneity of the HF population studied (in terms of age, demographics, comorbidities or HF severity), diverse sample sizes and divergent study design characteristics. Studies reporting differences in HRQOL among patients with HF based on EF are summarized in Table 3.

## 7. Differences in HRQOL Based on Geographic Location and Ethnicity

HRQOL has been evaluated in patients with HF from different parts of the world and has been found to demonstrate considerable variations among different geographic regions and different ethnic groups. Indeed, the G-CHF study, which is the largest study thus far to have systematically assessed HRQOL in a diverse population of patients with HF across the globe, has provided valuable insights into existing differences in HRQOL among various geographic regions with different income levels, diverse healthcare systems and varying levels of health literacy. The study enrolled 23,291 patients with HF from 40 countries across eight major geographic regions (North America, South America, Western Europe, Eastern Europe, Middle East, South Asia, East Asia and Africa) and measured HRQOL with the use of KCCQ-12. The mean KCCQ-12 summary score was 55 ± 0.2. After controlling for age, sex and HF severity, the lowest HRQOL scores were recorded in Africa (39.5 ± 0.3) and Eastern Europe (51.3 ± 0.6), whereas the highest HRQOL score was observed in Western Europe (62.5 ± 0.4). In all geographic regions, the most affected KCCQ-12 domain was the one reflecting general quality of life, while the least affected was the domain of symptom burden. Across all regions, HRQOL was found to be a strong and independent predictor of all-cause mortality and HF hospitalizations, irrespective of EF or NYHA functional class. The strongest association between HRQOL and adverse clinical outcomes was observed in Eastern Europe followed by the Middle East, whereas the weakest association was found in South Asia closely followed by South America and Africa [93]. 

Geographic variations in HRQOL were also evident in another large clinical trial, PARADIGM-HF. This trial included 8399 patients with HFrEF from 47 countries extending over the following geographic regions: North America, Latin America, Western Europe, Central/Eastern Europe/Russia and Asia/Pacific. HRQOL was assessed by means of the KCCQ clinical summary score. Patients from Central/Eastern Europe/Russia exhibited the lowest HRQOL at baseline, which was also accompanied by worse symptoms and signs and more severe functional limitation according to NYHA class. On the other hand, patients from Latin America and the Asia/Pacific region displayed the highest HRQOL. At 8-month follow-up, a higher proportion of patients from Central/Eastern Europe/Russia (32%) displayed deterioration in HRQOL, whereas considerably fewer patients from Latin America (24%) and Asia/Pacific (18%) experienced worsening of their HRQOL [109]. 

Another study aimed to investigate ethnic differences in HRQOL among patients with HFrEF by using a combined international cohort of 5697 patients from North America, Europe and Asia. For this purpose, participants from two clinical trials were used in the analysis: Heart failure: A controlled trial investigating outcomes in exercise training (HF-ACTION) and ASIAN-HF. Patients from HF-ACTION, originating from the USA, Canada and France, were divided into two ethnic groups: White and Black. Patients from ASIAN-HF, originating from 11 Asian countries, were divided into four ethnic groups: Chinese, Indian, Malay and Japanese/Korean. KCCQ was used for the measurement of HRQOL. Patients of Malay ethnicity had the worst HRQOL, followed by patients of Chinese ethnicity. On the contrary, Whites and Japanese/Korean had the highest KCCQ overall summary scores. Interestingly, in the self-efficacy subscale of the KCCQ, all Asian ethnicities scored significantly lower than Whites and Blacks [110].

Along the same lines, a prospective nationwide study from Singapore aimed to investigate ethnic differences in 1070 patients with HF, 62.3% of whom were of Chinese ethnicity, 26.7% of Malay ethnicity and 10.9% of Indian ethnicity. HRQOL was evaluated at baseline and at 6 months with the use of MLHFQ. At baseline, patients of Chinese ethnicity displayed better HRQOL than patients of Malay and Indian ethnicity, in both total MLHFQ score as well as in the MLHFQ physical and emotional dimension scores. More importantly, ethnicity remained a strong independent predictor of HRQOL even after adjusting for variables, such as NYHA class, NT-proBNP levels, comorbidities, medical treatment and demographic factors. At 6 months, HRQOL improved in all three ethnic groups, with the greatest improvement seen in Indians, so that ethnic disparities in HRQOL were no longer statistically significant [111]. 

A small-scale cross-sectional study compared HRQOL between American and Taiwanese patients with HF. HRQOL was measured by MLHFQ. American patients had significantly worse HRQOL than patients from Taiwan, both in the total MLHFQ score and in the physical dimension score. The investigators hypothesized that the better HRQOL in Taiwanese patients might be related to factors such as different family structure with higher levels of social support, less financial burden from medical care and lower activity levels. In detail, Taiwanese patients are more likely to receive support by another family member, since they tend to live alone less frequently than Americans. Moreover, the healthcare system in Taiwan provides more financial support to patients with HF, by entirely covering medical and healthcare costs through national health insurance. Lastly, Taiwanese have a more sedentary lifestyle and may not feel as functionally limited or disabled by their HF as American patients with HF who generally lead a more active life. Nevertheless, in both ethnic groups, symptom severity was the most significant predictor of HRQOL [112]. In addition, when HRQOL was compared between Canadian and American patients who were hospitalized due to acute decompensated HF and were enrolled in the acute study of clinical effectiveness of nesiritide in decompensated heart failure (ASCEND-HF) trial, it was found that HRQOL (measured with the use of EQ-5D) was similar between the two groups of patients at baseline (on hospital admission). Canadians reported more problems in the dimensions of mobility and self-care, whereas patients from the USA experienced more problems with pain and anxiety/depression. However, upon discharge as well as 30 days post-discharge, Canadians reported significantly better HRQOL than US patients [113].

In the CHAMP-HF registry, HRQOL was investigated with the use of KCCQ-12 in 3494 patients with HFrEF from 140 sites across the USA. In the overall cohort, the mean KCCQ overall summary score was 64.2 ± 23.9. Significant differences in HRQOL were noted among different ethnic groups. In unadjusted analyses, Blacks and Hispanics had worse KCCQ overall summary scores than Whites. After adjusting for sociodemographic factors, clinical characteristics and medical therapies, statistically significant differences remained only for Hispanics who continued to demonstrate worse HRQOL than Whites [114]. Notwithstanding, in an older study of 1212 patients with HF from the USA, no significant differences were observed in baseline MLHFQ scores among non-Hispanic Whites, non-Hispanic Blacks and Hispanics. In that study, it was also noted that HRQOL improved more over the course of time in Hispanics versus the other two ethnic groups [115]. On the other hand, in the telemonitoring to improve heart failure outcomes (Tele-HF) trial, a US multicenter, randomized controlled trial, data were analyzed from 1427 patients with HF who had been recently (in the previous month) hospitalized due to HF. Of the patients, 45% were non-Hispanic Black and 55% were non-Hispanic White. HRQOL was assessed with the use of KCCQ at baseline, as well as at 3 and 6 months. At baseline (shortly after hospital discharge), Black patients reported better HRQOL than Whites in almost all KCCQ domains, except for the subdomains of self-efficacy and symptom stability. However, no differences in HRQOL were observed between these two racial groups at 3- and 6-month follow-up [116]. 

The exact cause of observed differences in HRQOL among patients from different geographic regions and of different ethnicities is not known. It could be postulated that disparities related to the infrastructure, function and quality of healthcare systems from different parts of the world, as well as inequalities with regard to the allocation of health resources and the access of patients with HF to cardiovascular care services, may partly account for the observed differences. Methodological variations and imbalances in sample sizes of the studies could also be contributing to the observed discrepancies. Furthermore, differences in the severity of symptoms, diversities in self-care behaviors and heterogeneities in socioeconomic development, income levels and cultural practices among various racial and ethnic groups, coupled with divergent spirituality, ethnocultural values and perceptions regarding general attitude of life, health and quality of life, could partially explain existing differences in HRQOL [24,115,117,118,119,120,121,122]. 

## 8. Conclusions

Although it is well recognized that HRQOL is profoundly impaired in HF, significant variations have been reported among patients with HF depending on sex, age, NYHA functional class, EF and geographic location or ethnicity. Indeed, women tend to display poorer HRQOL compared to men. Moreover, most studies point towards the fact that younger patients with HF exhibit lower levels of HRQOL than older ones. It has also been consistently shown that patients in higher NYHA class (III–IV) have significantly more impaired HRQOL than those in a better NYHA functional class (I–II). Regarding differences in HRQOL based on EF, results have been contradictory so far, with most studies reporting worse HRQOL in patients with HFpEF compared to patients with HFrEF or HFmrEF. Based on geographic location, in the largest HRQOL study that has been conducted thus far, the lowest HRQOL levels have been recorded in Africa and Eastern Europe and the highest in Western Europe. Patients of Malay ethnicity have been found to exhibit poorer HRQOL when compared to other ethnicities from Asia, Europe and North America. In the comparison among White, Black and Hispanic ethnicities, inconclusive results have been reported.

Given that oftentimes contradictory results have arisen, there are still many unresolved issues with regard to the underlying differences that have been observed in various aspects of HRQOL among patients with HF. Thus, there is an ever-growing need for a strategic framework to be implemented in the assessment of HRQOL in patients with HF so as to achieve homogeneity in the process of evaluating and reporting HRQOL outcomes, eliminate any potential uncertainties, bridge existing differences whenever possible and reach more definite conclusions that will help optimize HF management and decision-making. In order to improve consistency and clarity, better refinement and consolidation of the HRQOL assessment methodology is required. Once a uniform, rigorous and standardized approach for measuring and reporting HRQOL is established, safe comparisons between different HRQOL measures can be made and firm and robust conclusions can be drawn [52,53,54]. 

Despite current methodological inconsistencies, it is obvious that differences in HRQOL exist among patients with HF. Accordingly, a thorough and in-depth understanding of the underlying differences may facilitate in increasing clinical awareness, modifying health-seeking behavior, informing management decisions and enhancing the quality of healthcare delivery. The ultimate goal is to mitigate any observed disparities, improve patient reported outcomes, identify optimal methods of patient monitoring and management and implement optimal treatment strategies tailored to the needs of each HF subpopulation. In order to gain such an understanding and achieve those goals, well-designed large-scale clinical trials with a focus on HRQOL in diverse populations with HF are warranted.

## Figures and Tables

**Table 1:** Studies evaluating sex-related differences in HRQOL among patients with heart failure.

Author Year	Study Design	Number of Patients	Men/Women	HRQOL Assessment Tool	Major Finding
Moradi et al., 2020 [24]	Systematic review and meta-analysis	3898	2174/1724	MLHFQ	HRQOL worse in women compared to men (pooled mean total MLHFQ score 45.6 vs. 40.7 respectively; *p* = 0.087).
Dewan et al., 2019 [57]	Analysis of dataset from 2 large randomized controlled HFrEF trials (PARADIGM-HF and ATMOSPHERE)	15,415 (HRQOL measured in 13,061)	12,058/3357	KCCQ	Women with HFrEF had worse HRQOL than men (median KCCQ clinical summary score 71.3 vs. 81.3; *p* < 0.0001). Largest difference in the domain of physical limitation.
Ravera et al., 2021 [58]	Post hoc analysis of a prospective cohort of HFrEF patients from BIOSTAT-CHF	1649	1276/373	KCCQ EQ-5D	Women with HFrEF had worse baseline HRQOL compared to men, both when assessed with KCCQ overall score (43.8 vs. 53.1; *p* < 0.001) and EQ-5D utility score (0.62 vs. 0.73; *p* < 0.001).
Garay et al., 2020 [59]	Pre-specified analysis of the VIDA-IC study	1028	719/309	KCCQ EQ-5D	HRQOL worse in women with HFrEF compared to men, both in the KCCQ overall summary score (54.7 ± 1.3 vs. 62.7 ± 0.8; *p* < 0.0001) and in the EQ-5D overall summary index (0.58 ± 0.01 vs. 0.67 ± 0.01; *p* < 0.0001).
Faxén et al., 2018 [60]	Retrospective analysis of data from the bi-national observational KaRen study	378	166/212	MLHFQ, EQ-5D-3L	HRQOL worse in women with HFpEF compared to men only in the EQ-5D-3L questionnaire (general HRQOL), both in its descriptive part (domains of mobility, usual activities and anxiety/depression) and in its EQ-VAS part (57 ± 20 in women vs. 61 ± 19 in men; *p* = 0.010). No significant difference in HF-specific HRQOL measured by MLHFQ (31 ± 21 in women vs. 29 ± 21 in men; *p* = 0.269).
Honigberg et al., 2020 [61]	Pooled secondary analysis of the RELAX and NEAT-HFpEF trials	323	158/165	MLHFQ	HRQOL did not differ between men and women with HFpEF (MLHFQ total score 46 ± 23.6 vs. 44 ± 22.3, respectively; *p* = 0.61).
Merrill et al., 2019 [62]	Post hoc, exploratory non-pre-specified subgroup analysis of TOPCAT-Americas trial	1767	885/882	KCCQ	Women with HFpEF (EF ≥ 45%) had worse HRQOL than men (KCCQ overall score 54.8 ± 22.5 vs. 61.4 ± 23.8, respectively; *p* < 0.001).
Dewan et al., 2019 [63]	Analysis of a pooled clinical trial cohort from CHARM-Preserved, I-Preserve and TOPCAT-Americas	8468	4010/4458	MLHFQ KCCQ	Women with HFpEF (EF ≥ 45%) had poorer HRQOL compared to men, as evidenced by lower (worse) median KCCQ clinical summary score in TOPCAT-Americas (56.3 [39.1–72.9] in women vs. 64.6 [45.8–82.3] in men; *p* < 0.001) and higher (worse) median MLHFQ score in I-Preserve and CHARM-Preserved (44 [29,30,31,32,33,34,35,36,37,38,39,40,41,42,43,44,45,46,47,48,49,50,51,52,53,54,55,56,57,58,59,60,61] in women vs. 37 [22,23,24,25,26,27,28,29,30,31,32,33,34,35,36,37,38,39,40,41,42,43,44,45,46,47,48,49,50,51,52,53,54] in men; *p* < 0.001).
Fonseca et al., 2021 [64]	Real-world cross-sectional study	804	517/287	MLHFQ, EQ-5D-5L	HRQOL worse in women with HF and EF ≤ 60% compared to men, as evidenced by higher (worse) overall MLHFQ mean score in women vs. men (37.9 vs. 34.6, respectively; *p* = 0.0481) and lower (worse) mean EQ-5D utility score (0.69 vs. 0.75, respectively; *p* = 0.0046) and EQ-VAS score (55.4 vs. 61.3, respectively; *p* < 0.0001).
Ma et al., 2022 [65]	Single-center prospective longitudinal study	154	94/60	KCCQ, EQ-5D	At baseline, HRQOL was similar between men and women with HF, both when assessed with KCCQ and EQ-5D. However, at 1, 6 and 12 months, women had statistically worse HRQOL compared to men in all scores (KCCQ, EQ-5D index, EQ-VAS).
Truby et al., 2020 [66]	Secondary analysis of the PAL-HF trial	150	79/71	KCCQ	Women with advanced HF had worse HRQOL than men, as evidenced by lower baseline KCCQ score (24.5 vs. 36.2, respectively; *p* = 0.04). Even after palliative care intervention, women’s HRQOL remained lower than that of men.
Blumer et al., 2021 [67]	Secondary analysis of the ASCEND-HF trial	7141	4697/2444	EQ-5D	Women with acute decompensated HF had worse HRQOL than men at all timepoints throughout hospitalization and post-discharge in both EQ-5D utility score and EQ-VAS score (all *p* ≤ 0.002).

Abbreviations: ASCEND-HF = Acute study of clinical effectiveness of nesiritide in decompensated heart failure; ATMOSPHERE = Aliskiren trial to minimize outcomes in patients with heart failure; BIOSTAT-CHF = A systems biology study to tailored treatment in chronic heart failure; CHARM-Preserved = Candesartan in heart failure: Assessment of reduction in mortality and morbidity-Preserved; EF = Ejection fraction; EQ-5D = Euro-Qol 5-dimensional questionnaire; EQ-5D-3L = Euro-Qol 5-dimensional 3-level questionnaire; EQ-5D-5L = Euro-Qol 5-dimensional 5-level questionnaire; EQ-VAS = Euro-Qol Visual Analogue Scale; HF = Heart failure; HFpEF = Heart failure with preserved ejection fraction; HFrEF = Heart failure with reduced ejection fraction; HRQOL = Health-related quality of life; I-Preserve = Irbesartan in heart failure with preserved ejection fraction; KaRen = Karolinska Rennes; KCCQ = Kansas City Cardiomyopathy Questionnaire; MLHFQ = Minnesota Living with Heart Failure Questionnaire; NEAT-HFpEF = Nitrate’s effect on activity tolerance in heart failure with preserved ejection fraction; PAL-HF = Palliative care in heart failure; PARADIGM-HF = Prospective comparison of angiotensin receptor neprilysin inhibitor (ARNI) with an angiotensin converting enzyme inhibitor (ACE-I) to determine impact on global mortality and morbidity in heart failure; RELAX = Phosphodiesterase-5 inhibition to improve clinical status and exercise capacity in heart failure with preserved ejection fraction; TOPCAT = Treatment of preserved cardiac function heart failure with an aldosterone antagonist; VIDA-IC = Quality of life and heart failure in Spain: Current situation; vs. = versus.

**Table 2:** Studies evaluating age-related differences in HRQOL among patients with heart failure.

Author Year	Study Design	Number of Patients	HRQOL Assessment Tool	Major Finding
Wong et al., 2013 [74]	Secondary analysis of the CHARM study	7599	MLHFQ	Patients were grouped into 5 age categories: 20–39 (n = 120), 40–49 (n = 538), 50–59 (n = 1527), 60–69 (n = 2395) and ≥70 years (n = 3019). HRQOL was worse in the youngest patients and improved with increasing age. Mean MLHFQ score was 52.6 in the age group 20–39 and decreased (improved) with increasing age: 50.8 (ages 40–49), 47.1 (ages 50–59), 38.9 (ages 60–69) and 35.3 (ages ≥ 70); *p* < 0.0001.
Reddy et al., 2020 [75]	Secondary analysis of RELAX, NEAT-HFpEF and INDIE-HFpEF trials	408	KCCQ MLHFQ	Patients with HFpEF belonging to the group with the worst HRQOL (MLHFQ score >57 in RELAX or KCCQ score ≤45 in NEAT-HFpEF and INDIE-HFpEF) were the youngest and had the highest BMI, the highest prevalence of obesity and diabetes mellitus and the lowest NT-proBNP levels.
Tromp et al., 2019 [76]	Retrospective analysis of TOPCAT-Americas, I-Preserve and CHARM-Preserved	8468	KCCQ MLHFQ	Patients with HFpEF (EF ≥ 45%) were stratified into 5 age categories: ≤55 (n = 522), 56–64 (n = 1679), 65–74 (n = 3405), 75–84 (n = 2464) and ≥85 years (n = 398). HRQOL (expressed by the KCCQ score in TOPCAT-Americas and MLHFQ score in I-Preserve and CHARM-preserved) was worse in younger patients compared to older ones. This association between HRQOL and age remained significant after correction for sex, history of atrial fibrillation, diabetes and BMI.
Tromp et al., 2018 [77]	Multinational, multicenter prospective study from Asia	1203	KCCQ	Patients with HFpEF from 11 Asian regions were grouped into 4 categories: very young (<55 years; n = 157), young (55–64; n = 284), older (65–74; n = 355) and elderly (≥75 years; n = 407). HRQOL was better in the very young compared to the elderly, as evidenced by better KCCQ scores for both the individual components and the overall and clinical summary scores.
Lawson et al., 2023 [78]	Analysis of data from the Swedish Heart Failure Registry	23,553	EQ-VAS	Patients were grouped into 5-year categories as follows: ≤60, 61–65, 66–70, 71–75, 76–80, 81–85 and >85 years old. Median EQ-VAS was higher (better HRQOL) in the youngest (70 [50,51,52,53,54,55,56,57,58,59,60,61,62,63,64,65,66,67,68,69,70,71,72,73,74,75,76,77,78,79,80]) compared to the oldest group (60 [50,51,52,53,54,55,56,57,58,59,60,61,62,63,64,65,66,67,68,69,70,71,72,73,74,75]). Although HRQOL improved from the youngest category up to the age category of 66–70, it worsened thereafter, since each increase in age category was accompanied by a gradual decrease (worsening) in EQ-VAS score, which was consistent across all EF ranges.
Gallagher et al., 2016 [79]	Prospective, cross-sectional study	104	MLHFQ	Age independently predicted HRQOL in community-dwelling patients with HF and a mean age of 80.93 years. Younger patients had worse HRQOL in all MLHFQ domains (physical, emotional, overall) than older ones.
Wang et al., 2022 [80]	Observational cohort study with analysis of prospectively collected data	1911	MLHFQ	Younger age was a predictor of worse HRQOL in community-dwelling patients with a mean age of 79 years who had a recent HF hospitalization. Younger patients had higher overall MLHFQ scores (worse HRQOL).
Masoudi et al., 2004 [81]	Multicenter prospective cohort study	484	KCCQ	At baseline, older patients (≥65 years) had better HRQOL than younger ones (<65 years), as evidenced by a higher mean KCCQ score (60 ± 25 vs. 54 ± 28, respectively; *p* = 0.005). However, at follow-up, among patients who experienced a deterioration in NYHA functional status, older patients suffered statistically significant declines in their KCCQ scores (−14.4 ± 22 points), whereas younger ones had no significant changes (+0.3 ± 18 points; *p* for age comparison = 0.0003).
Chernomordik et al., 2017 [82]	Single-center longitudinal cohort study	287	MLHFQ	At baseline, younger patients with advanced HF and refractory symptoms were more likely to have worse HRQOL. However, after one year of treatment with intermittent low-dose inotropes, younger age was an independent predictor of improvement in HRQOL.
Moser et al., 2013 [83]	Observational, cross-sectional study	603	MLHFQ	Patients with HF were divided into 4 age groups: ≤53, 54–62, 63–70, ≥71 years. HRQOL was worse in the youngest group and best in the two oldest groups. The youngest group also had higher levels of anxiety and depression.

Abbreviations: BMI = Body mass index; CHARM = Candesartan in heart failure: Assessment of reduction in mortality and morbidity; CHARM-Preserved = Candesartan in heart failure: Assessment of reduction in mortality and morbidity-Preserved; EF = Ejection fraction; EQ-VAS = Euro-Qol Visual Analogue Scale; HF = Heart failure; HFpEF = Heart failure with preserved ejection fraction; HRQOL = Health-related quality of life; INDIE-HFpEF = Inorganic nitrite delivery to improve exercise capacity in heart failure with preserved ejection fraction; I-Preserve = Irbesartan in heart failure with preserved ejection fraction; KCCQ = Kansas City Cardiomyopathy Questionnaire; MLHFQ = Minnesota Living with Heart Failure Questionnaire; NEAT-HFpEF = Nitrate’s effect on activity tolerance in heart failure with preserved ejection fraction; NT-proBNP = N-terminal pro-brain natriuretic peptide; NYHA = New York Heart Association; RELAX = Phosphodiesterase-5 inhibition to improve clinical status and exercise capacity in heart failure with preserved ejection fraction; TOPCAT = Treatment of preserved cardiac function heart failure with an aldosterone antagonist; vs. = versus.

**Table 3:** Studies evaluating differences in HRQOL among patients with heart failure based on ejection fraction.

Author Year	Country	Number of Patients	HF Type	HRQOL Assessment Tool	Major Finding
Chen et al., 2019 [92]	China	841	HFrEF, HFmrEF, HFpEF	MLHFQ	HRQOL worse in HFrEF patients (total MLHFQ score 43.1) vs. HFmrEF (36.9) and HFpEF (33.2) patients; *p* < 0.001.
Johansson et al., 2021 [93]	40 countries from 8 world regions	23,291	HFrEF (EF < 40%), HF with EF ≥ 40%	KCCQ-12	HRQOL worse in HFrEF patients (KCCQ-12-SS 52.8 ± 0.2) vs. patients with HF with EF ≥ 40% (54.6 ± 0.3); *p* < 0.0001.
Gastelurrutia et al., 2018 [94]	Spain	1405	HFrEF, HFmrEF, HFpEF	MLHFQ	HRQOL better in HFmrEF patients (mean MLHFQ score 30.1 ± 18.3) than in HFpEF patients (36.5 ± 20.7; *p* = 0.003) and similar to HFrEF patients (30.8 ± 18.5; *p* = 0.61).
Lawson et al., 2023 [78]	Sweden	23,533	HFrEF, HFmrEF, HFpEF	EQ-VAS	HRQOL worse in HFpEF patients (median EQ-VAS 62 [50,80]) vs. HFmrEF (70 [50,80]) and HFrEF (65 [50,80]) patients.
Streng et al., 2018 [95]	11 European countries	3499	HFrEF, HFmrEF, HFpEF	KCCQ, EQ-VAS	HRQOL worse in HFpEF patients (median KCCQ overall score 38 [24,25,26,27,28,29,30,31,32,33,34,35,36,37,38,39,40,41,42,43,44,45,46,47,48,49,50,51,52,53]) vs. HFmrEF (43 [30,31,32,33,34,35,36,37,38,39,40,41,42,43,44,45,46,47,48,49,50,51,52,53,54,55,56,57,58,59]) and HFrEF (47 [31,32,33,34,35,36,37,38,39,40,41,42,43,44,45,46,47,48,49,50,51,52,53,54,55,56,57,58,59,60,61,62,63,64]) patients; *p* < 0.001.
Bekfani et al., 2021 [96]	Germany	55	HFrEF (EF < 40%), HFpEF (EF ≥ 50%)	SF-36, EQ-VAS	HRQOL worse in HFpEF patients vs. HFrEF patients in the MCS (43.6 ± 7.1 vs. 50.2 ± 10) and VT (47.5 ± 8.4 vs. 53.6 ± 8.6) scores of the SF-36; *p* < 0.05.
Warraich et al., 2018 [97]	USA	202	HFrEF (EF < 45%), HFpEF (EF ≥ 45%)	KCCQ, SF-12, EQ-5D-5L	HRQOL worse in HFpEF patients vs. HFrEF patients with all 3 assessment tools.
Lewis et al., 2007 [98]	USA and Canada	2709	HFrEF (EF ≤ 40%), HFpEF (EF > 40%)	MLHFQ	No difference in mean MLHFQ summary score between HFrEF (40.8) and HFpEF (41.1) patients; *p* = 0.67. MLHFQ physical score slightly worse in HFpEF (19.4) vs. HFrEF (18.5) patients; *p* = 0.04.
Hoekstra et al., 2011 [99]	Netherlands	290	HFrEF (EF < 40%), HFpEF (EF ≥ 40%)	MLHFQ, RAND-36, Cantril’s Ladder of Life	No difference in HRQOL between HFpEF and HFrEF patients.
Sepehrvand et al., 2020 [100]	Canada	360	HFrEF (EF < 45%), HFpEF (EF ≥ 45%)	KCCQ, EQ-5D-5L, FACT-An	No statistically significant differences in HRQOL between HFpEF and HFrEF patients.
Rickenbacher et al., 2017 [101]	Switzerland and Germany	622	HFrEF, HFmrEF, HFpEF	MLHFQ, SF-12	No significant differences in HRQOL among patients with HFrEF, HFmrEF and HFpEF.
Ahmeti et al., 2017 [102]	Kosovo	118	HFrEF, HFpEF	MLHFQ	No difference in HRQOL between HFpEF and HFrEF patients.
Jorge et al., 2017 [103]	Brazil	59	HFrEF, HFpEF	SF-36	No difference in HRQOL between HFpEF and HFrEF patients.
Chandra et al., 2019 [104]	International	11,622	HFrEF (EF ≤ 40%), HFpEF (EF ≥ 45%)	KCCQ	In unadjusted models, KCCQ overall summary score was worse in HFpEF (71.4 ± 18.9) than HFrEF (72.7 ± 19.5) patients; *p* < 0.001. However, after adjustment, HRQOL was similar between the 2 groups.
Zamora et al., 2022 [105]	Spain	1040	HFrEF, HFimpEF	MLHFQ	HRQOL similar between patients with HFimpEF and patients with HFrEF who did not fulfill criteria for improved EF at 1-year follow-up.
Wohlfahrt et al., 2021 [106]	USA	319	HFrEF, HFpEF, HFimpEF	KCCQ VAS PROMIS	Patients with HFimpEF (EF recovered to 50% or more) showed significant improvement in HRQOL at 1 year, whereas HFrEF patients had much smaller improvement and HFpEF patients no significant improvement.
DeVore et al., 2022 [107]	USA	1690	HFrEF, HFimpEF	KCCQ-12	Patients with HFimpEF showed significantly greater improvement in HRQOL at follow-up compared to HFrEF patients without improvement of ≥10% in EF.
Joyce et al., 2016 [108]	USA	726	HFrEF (persistent EF < 50%), HFpEF (EF ≥ 50%), HFimpEF (EF recovered to 50% or more)	VAS	Patients with HFimpEF had the highest overall HRQOL scores among the 3 groups and significantly better breathing VAS scores than HFpEF patients.

Abbreviations: EF = Ejection fraction; EQ-5D-5L = Euro-Qol 5-dimensional 5-level questionnaire; EQ-VAS = Euro-Qol Visual Analogue Scale; FACT-An = Functional assessment of cancer therapy-anemia questionnaire; HF = Heart failure; HFimpEF = Heart failure with improved ejection fraction; HFmrEF = Heart failure with mildly reduced ejection fraction; HFpEF = Heart failure with preserved ejection fraction; HFrEF = Heart failure with reduced ejection fraction; HFrecEF = Heart failure with recovered ejection fraction; HRQOL = Health-related quality of life; KCCQ = Kansas City Cardiomyopathy Questionnaire; KCCQ-12: 12-item Kansas City Cardiomyopathy Questionnaire Short Version; KCCQ-12-SS = 12-item Kansas City Cardiomyopathy Questionnaire summary score; MCS = Mental component score; MLHFQ = Minnesota Living with Heart Failure Questionnaire; PROMIS = Patient-reported outcomes measurement information system; SF-12 = 12-item Short Form Health Survey questionnaire; SF-36 = Medical outcomes study 36-item Short Form Health Survey questionnaire; USA = United States of America; VAS = Visual analogue scale; vs. = Versus; VT = Vitality.

## Data Availability

Not applicable.
